# Assessment of salivary alpha-amylase and cortisol as a pain related stress biomarker in dogs pre-and post-operation

**DOI:** 10.1186/s12917-021-03114-2

**Published:** 2022-01-13

**Authors:** Eun-Ha Kang, Seol-Hee Park, Ye-In Oh, Kyoung-Won Seo

**Affiliations:** 1 Department of Veterinary Internal Medicine, College of Veterinary Medicine, Chungnam National University, Daejeon, 34134 Republic of Korea; 2 Laboratory of Veterinary Internal Medicine, The research Institute for Veterinary Science, College of Veterinary Medicine, Seoul National University, Seoul, 08826 Republic of Korea

**Keywords:** Alpha-amylase, Canine, Glasgow composite measure pain scale (CMPS-SF), Cortisol, Pain, Stress

## Abstract

**Background:**

The use of salivary biomarkers has garnered attention because the composition of saliva reflects the body’s physiological state. Saliva contains a wide range of components, including peptides, nucleic acids, electrolytes, enzymes, and hormones. It has been reported that salivary alpha-amylase and cortisol are biomarkers of stress related biomarker in diseased dogs; however, evaluation of salivary alpha-amylase and cortisol pre- and post- operation has not been studied yet. The aim of this study was to evaluate salivary alpha-amylase and cortisol levels in dogs before and after they underwent surgery and investigate the association between the salivary alpha-amylase and cortisol activity and pain intensity. For this purpose, a total of 35 dogs with disease-related pain undergoing orthopedic and soft tissue surgeries were recruited. Alpha-amylase and cortisol levels in the dogs’ saliva and serum were measured for each using a commercially available canine-specific enzyme-linked immunosorbent assay kit, and physical examinations (measurement of heart rate and blood pressure) were performed. In addition, the dogs’ pre- and post-operative pain scores determined using the short form of the Glasgow Composite Measure Pain Scale (CMPS-SF) were evaluated.

**Results:**

After surgery, there was a significant decrease in the dogs’ pain scores (0.4-fold for the CMPS-SF, *p* < 0.001) and serum cortisol levels (0.73-fold, *p* < 0.01). Based on their pre-operative CMPS-SF scores, the dogs were included in either a high-pain-score group or a low-pain-score group. After the dogs in the high-pain-score group underwent surgical intervention, there was a significant decrease in their CMPS-SF scores and levels of salivary alpha-amylase, serum alpha-amylase, and serum cortisol. Additionally, there was a positive correlation between salivary alpha-amylase levels and CMPS-SF scores in both the high- and low-pain-score groups.

**Conclusions:**

The measurement of salivary alpha amylase can be considered an important non-invasive tool for the evaluation of pain-related stress in dogs.

**Supplementary Information:**

The online version contains supplementary material available at 10.1186/s12917-021-03114-2.

## Background

Saliva is a protein-rich fluid secreted from major paired salivary glands, such as the parotid, zygomatic, mandibular, and sublingual glands [1]. It also contains molecules that are present in the blood [2]. Some molecules enter saliva via passive diffusion, active transportation, and ultrafiltration from blood. A wide range of molecules present in saliva, including peptides, nucleic acids, electrolytes, enzymes, and hormones, are measurable [3].

The use of salivary biomarkers has garnered attention because saliva contains constituents that reflect the physiological state of the body [4], and information regarding the amount and nature of such constituents can easily be obtained through non-invasive means [5]. Salivary alpha-amylase and cortisol have been suggested as stress biomarker in human in several studies [5, 6]. Although, salivary alpha-amylase was found to increase with disease related stress in dogs [7, 8], to the best of our knowledge, no attempt has been made yet to evaluate salivary alpha-amylase and cortisol activity compared to pain scale score pre- and post operation in dogs.

In veterinary clinics, several pain-assessment tools, such as simple descriptive scores, visual analogue scales, numerical rating scales, the Glasgow Composite Measure Pain Scale (CMPS), and the short form of the Glasgow CMPS (CMPS-SF) have been used [9–12]. Although these scales were based on scientific observations, dogs are non-verbal and must rely on perceptions and interpretations of behavior to assess pain intensity. Such recognition and interpretation can be influenced by the subjective judgment of clinicians. Consequently, there is a need for complementary tools that can be used for objective assessments of stress and pain in dogs. The purpose of this study was to evaluate salivary alpha-amylase and cortisol levels in diseased dogs before and after they underwent surgery (of different types); additionally, we aimed to investigate the association between alpha-amylase and cortisol activity and pain related stress.

## Results

### Characteristics of study population

A total of 35 client-owned dogs in clinical cases involving dogs that were scheduled to undergo orthopedic or major soft tissue surgery were included. Their ages ranged from 1 to 17 years (mean age: 7.6 years); 21 dogs were males, 19 of which had been castrated, and 14 dogs were females, 8 of which had been spayed. The underlying conditions due to which the dogs underwent surgery were orthopedic conditions (*n* = 16), tumors (*n* = 6), pyometra (*n* = 5), renal calculi (*n* = 3), injury caused by car accidents (n = 3), and portosystemic shunts (*n* = 2) (Table 1). No dog has a history of pain medication prior to surgical intervention. To control surgery related pain, analgesics were provided some period after the surgery. Most of dogs were withdrawn pain medication within 3–4 days of surgery and the longest duration for analgesic required was 5 days. For that reason, 7 days after the surgery was set as the post-sample time. No analgesics were administered at the time of post-operative sampling.

**Table 1:** Patient signalment

Variables	Total (***n*** = 35)
**Signalment**	
Age (years)	7.59 ± 4.8
Sex and neutered status	
Male not neutered	2
Male neutered	19
Female not neutered	6
Female neutered	8
**Underlying illness**	
Medial patella luxation	7
Cranial cruciate ligament rupture	4
Bone fracture	5
Tumor	6
Pyometra	5
Renal calculus	3
Hit by car	3
Portosystemic shunt	2

### Changes in stress-parameter values after surgery

**Fig. 1:**
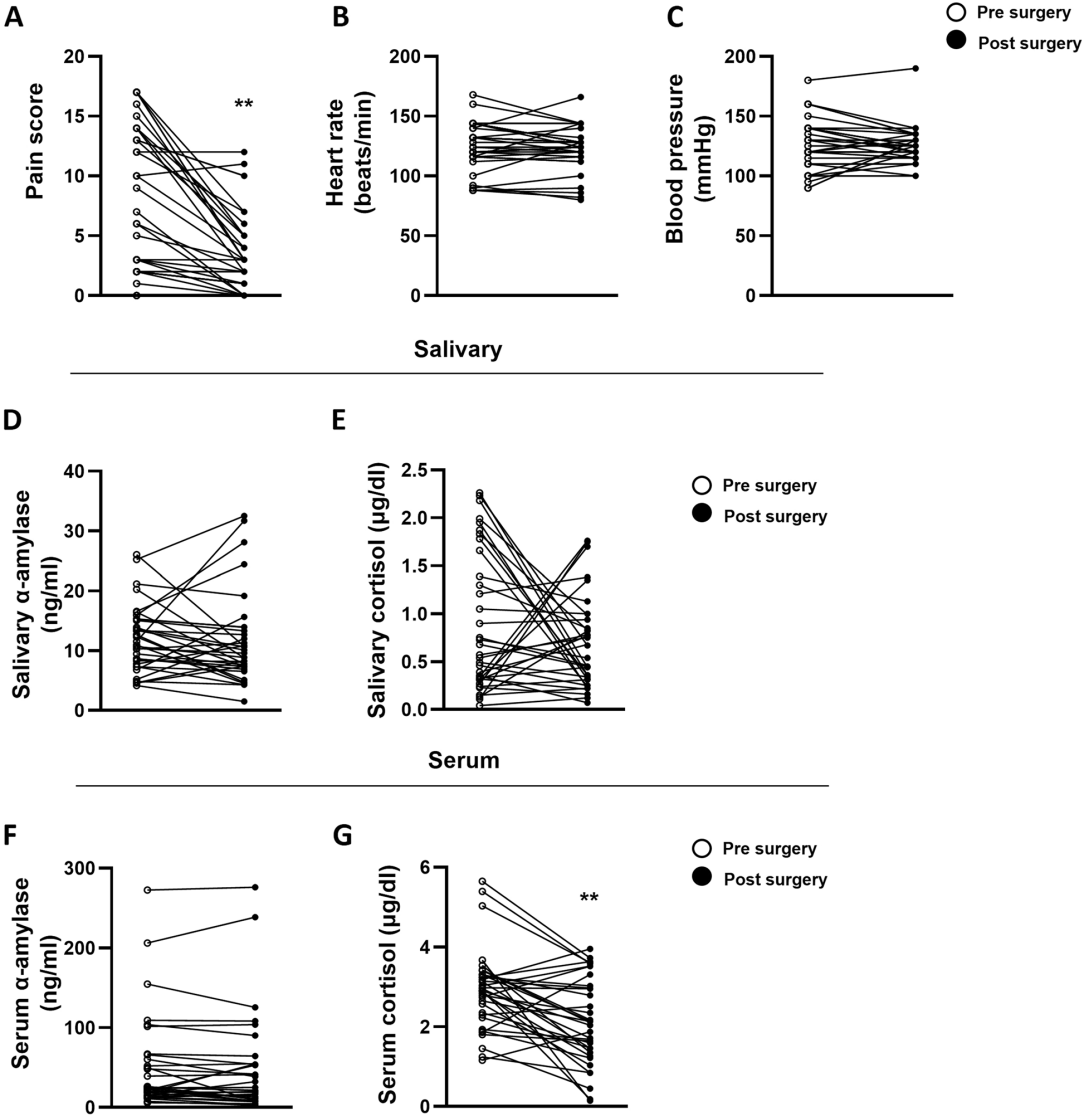
Changes in stress-parameter values in dogs who underwent surgery. Stress parameters were evaluated before and 7 days after the surgery. Pain score and serum cortisol levels were significantly decreased after the surgery. **A** Pain score **B** Heart rate **C** Blood pressure **D**, **E** α-amylase and cortisol levels from saliva **F**, **G** α-amylase and cortisol levels from serum. (**) values are significantly different (*p* < 0.01)

Figure 1 shows the changes in stress-related parameters in dogs that underwent surgery. Pain assessment was performed using the CMPS-SF, and pre-operative and post-operative values of other stress-related variables such as heart rate (HR), blood pressure (BP), and levels of salivary alpha-amylase, salivary cortisol, serum alpha-amylase, and serum cortisol were compared. Postoperatively, there was a decrease in the CMPS-SF scores of 31 of the 35 dogs, which suggests that pain was attenuated by treatment of underlying conditions through surgery. Based on their preoperative CMPS-SF scores, the dogs were included in either a high-pain-score group or a low-pain-score group. In both groups, pain scores and serum cortisol levels decreased after surgery (Table 2). In high pain group, CMPS-SF score was decreased 0.43-fold (*p* < 0.001) and serum cortisol was decreased 0.76-fold (*p* = 0.02). In low pain group, CMPS-SF score was decreased 0.36-fold (*p* = 0.001) and serum cortisol was decreased 0.73-fold (*p* < 0.01). Interestingly, among the dogs in the high-pain-score group, with pain relief after surgery, their salivary and serum alpha-amylase levels were decreased by 0.76-fold (*p* = 0.03) and 0.87-fold (*p* = 0.015), respectively (Table 2). With the treatment of underlying conditions through surgery, there was a decrease in physiological stress, as indicated by decreased serum cortisol levels. HR and BP levels were not different after the surgery (Fig. 1).

**Table 2:** Stress related parameters before and after surgery

Variables	High pain (***n*** = 15)	Low pain (***n*** = 20)
**Before Surgery**		
CMPS-SF	14.27 ± 2.19	3.05 ± 2.5
Heart rate (beats/min)	125.29 ± 24.75	123.58 ± 17.02
Blood pressure (mmHg)	125.36 ± 22.91	120.26 ± 20.24
Salivary α-amylase (ng/ml)	14.87 ± 5.85	9.80 ± 4.33
Serum α-amylase (ng/ml)	49.57 ± 43.42	50.82 ± 70.85
Salivary cortisol (mcg/dl)	0.82 ± 0.61	0.99 ± 0.81
Serum cortisol (mcg/dl)	2.85 ± 0.56	3.13 ± 1.16
**After surgery**		
CMPS-SF	6.20 ± 3.21 ***	1.11 ± 1.15 **
Heart rate (beats/min)	122.43 ± 21.53	122.84 ± 16.59
Blood pressure (mmHg)	122.14 ± 23.51	123.42 ± 8.83
Salivary α-amylase (ng/ml)	11.35 ± 6.83 *	10.16 ± 6.85
Serum α-amylase (ng/ml)	42.98 ± 40.34 *	50.21 ± 76.84
Salivary cortisol (mcg/dl)	0.66 ± 0.39	0.717 ± 0.534
Serum cortisol (mcg/dl)	2.15 ± 1.04 *	2.28 ± 1.07 **

Data are mean ± SD

High pain score group: pre-operative CMPS-SF > 9; Low pain score group: pre-operative CMPS-SF ≤9 pain score

CMPS-SF; short form of the Glasgow composite measure pain scale

* Significance of differences in stress parameters before and after surgery. *P < 0.05 **P < 0.001 ***P < 0.0001

### Validity of salivary alpha-amylase and cortisol as biomarkers of pain-related stress

**Fig. 2:**
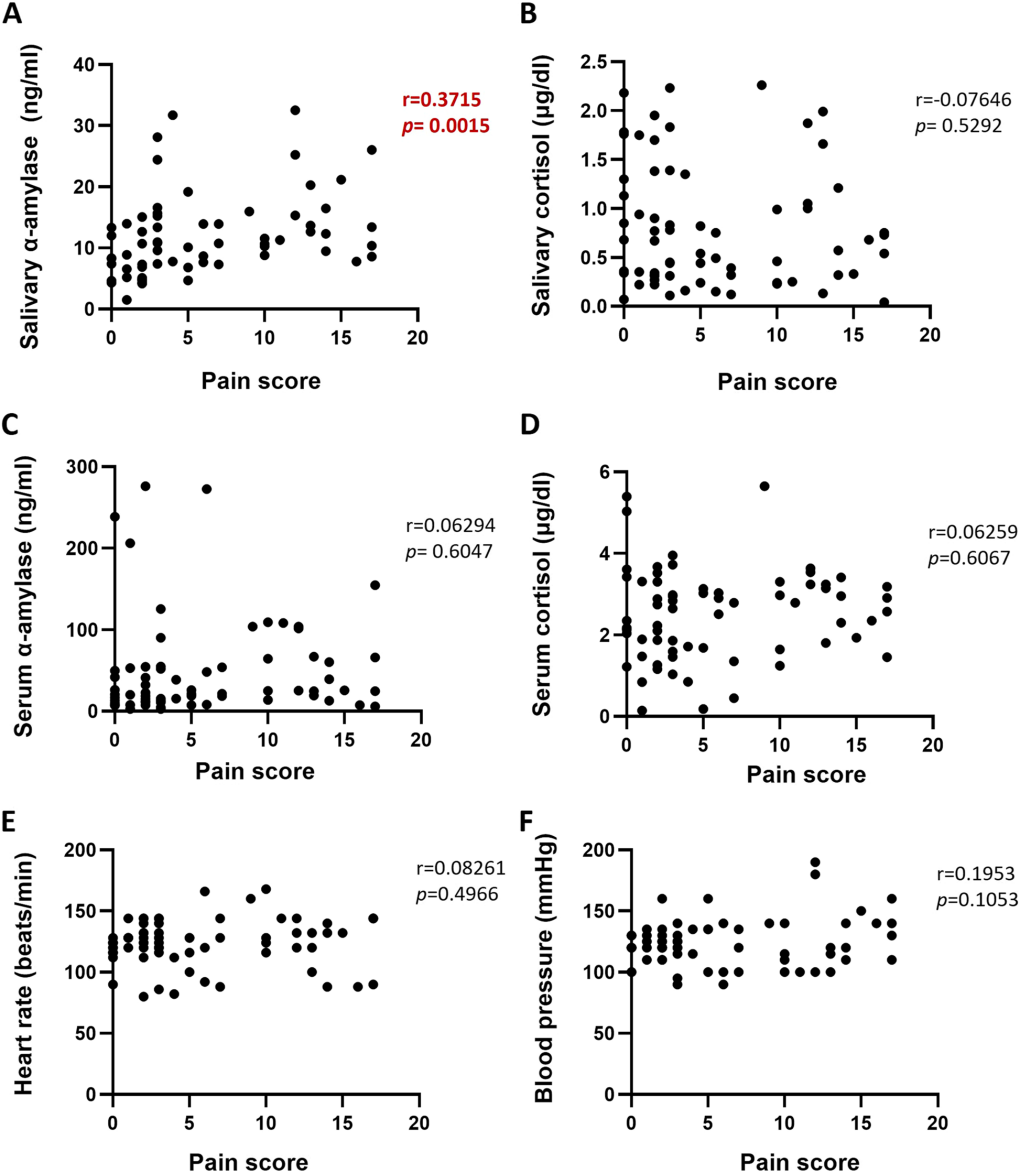
Scatter plot depicting the correlation between salivary alpha-amylase levels and pain scores. Scatter plot between pain score with other stress related parameters: **A** salivary α-amylase **B** Salivary cortisol **C** Serum α-amylase **D** Serum cortisol **E** Heart rate **F** Blood pressure. *p* value < 0.05 was considered significant

Figure 2 depicts a moderate positive correlation between salivary alpha-amylase levels and CMPS-SF scores (r = 0.3715, *p* = 0.0015). Additionally, it was found that there was a correlation between salivary alpha-amylase levels and CMPS-SF scores among both the high- and low-pain-score groups (r = 0.3375, *p* = 0.038 and r = 0.3715, p = 0.0015, respectively) (Supplementary Figs. 1A and 1B). With respect to serum cortisol levels, the correlation between serum cortisol levels and CMPS-SF scores was not statistically significant (r = 0.3493, *p* = 0.058) (Supplementary Fig. 2).

### Association between salivary and serum stress markers

**Fig. 3:**
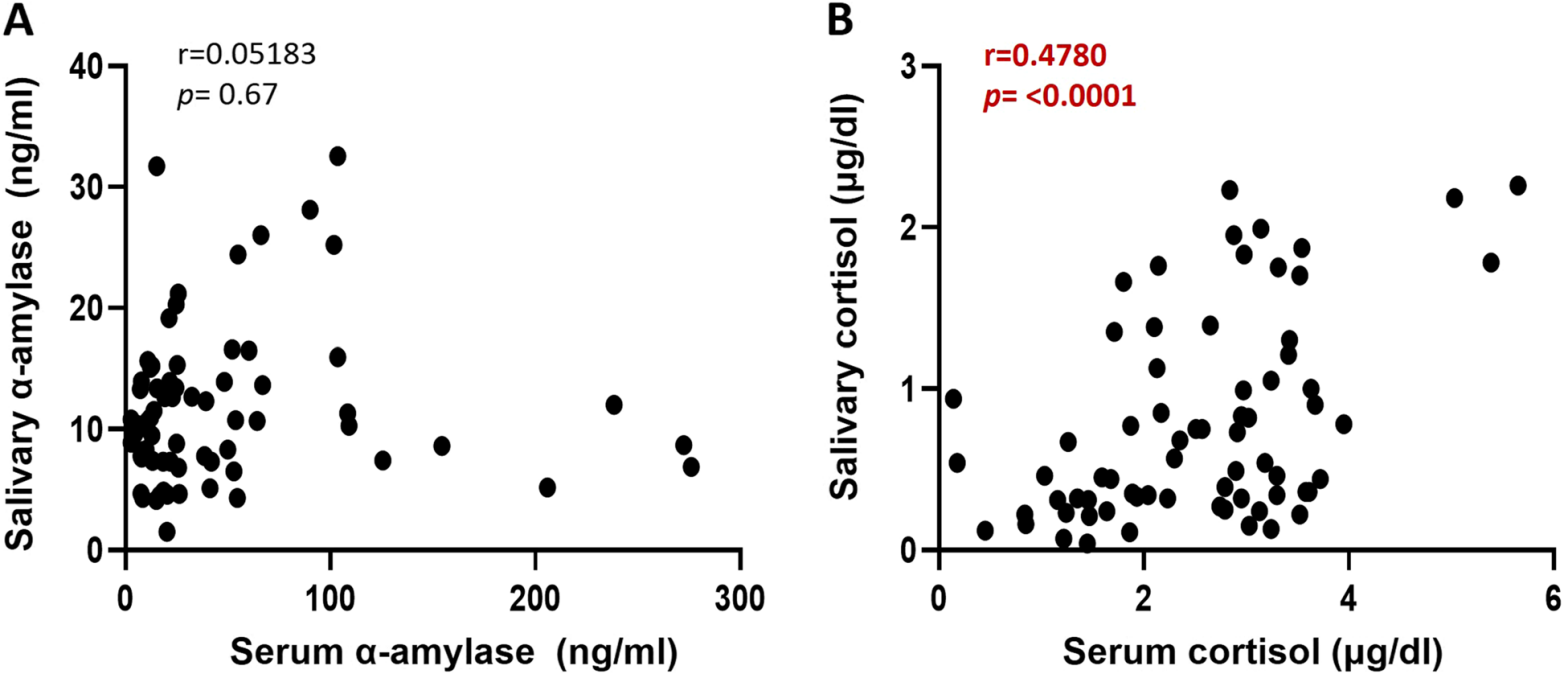
Correlation between salivary and serum cortisol levels. **A**. Scatter plot between salivary α-amylase with serum α-amylase **B**. Scatter plot between salivary cortisol with serum cortisol. *p* value < 0.05 was considered significant

There was no correlation between salivary alpha-amylase levels and serum alpha-amylase levels. However, the findings revealed a significant correlation between salivary cortisol and serum cortisol levels (r = 0.4780, *p* < 0.0001) (Fig. 3).

## Discussion

This study was performed with the objective of evaluating whether levels of salivary biomarkers such as alpha-amylase and cortisol reflect pain-related stress in dogs that undergo surgery and are expected to experience pain-related stress before they undergo surgical interventions. Pain is a collection of emotional and sensory perception as a results of the activation of nociceptive pathways following noxious stimuli [13]. Stress and pain are distinct yet overlapping phenomena that are inexorably linked to each other [14, 15]. Physical and psychological stimuli lead to the activation of the hypothalamic–pituitary–adrenal (HPA) axis, leading to the release of glucocorticoids [16], which also activates the autonomic nervous system, leading to the release of catecholamines [17]. Researchers have been attempting to develop effective tools to measure pain intensities and associated stress statuses [18–20], which can be used to monitor pain management during treatment.

The CMPS-SF used in this study is a modified form of the Glasgow CMPS; the CMPS-SF was developed for routine clinical use, which involves an emphasis on speed and ease of use [11]. Our data showed that after they had received surgical treatment, most of the included dogs had decreased CMPS-SF scores. This result was expected because the treatment of diseases leads to behavioral changes and reduction in pain [10]. An important finding in the present study was the finding regarding the correlation between serum alpha-amylase levels and CMPS-SF scores. The use of salivary alpha-amylase levels as an index for the assessment of pain intensity in humans has received much attention [21], and it has been suggested in some studies that salivary alpha-amylase levels can be used effectively to monitor pain in patients who cannot perform self-assessments [22, 23]; in veterinary practice, questionnaires cannot be administered to patients for the assessment of subjectively perceived stress, which is a critical impediment to the measurement of stress. Therefore, our findings suggest that the measurement of salivary alpha-amylase levels may be an effective tool for the assessment of pain-related stress in dogs. In the present study, we found that the difference between pre-and post-operative levels of salivary and serum alpha-amylase was significant only in the high-pain-score group. The significant postoperative decrease in salivary and serum alpha-amylase levels in the high-pain-score group suggests that psychological factors induced by significant pain contributed to the relatively higher pre-operative salivary and serum alpha-amylase levels. In all groups, there was a post-operative decrease in serum cortisol levels. In a recent study in which salivary alpha-amylase and cortisol levels in healthy human volunteers who were exposed to the Trier Social Stress Test and electric simulation stress were measured, it was found that salivary alpha-amylase levels displayed a rapid increase and returned to baseline levels 20 min after exposure to the stress challenge; in contrast, salivary cortisol levels showed a delayed increase, remaining significantly elevated from baseline levels 20 min after exposure to stress [24]. In this regard, it can be suspected that in the present study, pain-related stress stimuli in the low-pain-score group were intermittent and weaker than those in the high-pain-score group, which may have led to the relatively lower salivary alpha-amylase levels in the low-pain-score group. A possible mechanism underlying high levels of alpha-amylase is that severe disease-related pain may cause increased psychological stress, which further activates the sympathetic–adrenal–medullary (SAM) system [25, 26]. This was evidenced by postoperatively reduced salivary alpha-amylase levels in the high-pain-score group.

Another interesting finding of this study is that no correlation was identified between serum alpha-amylase and salivary alpha-amylase levels; however, there was a significant correlation between salivary cortisol and serum cortisol levels. These results point towards the differences in the mechanisms of production and release of cortisol and alpha amylase. Cortisol is produced by the adrenal glands in response to the activation of the HPA axis. Bozovic et al. reported that cortisol responses lag behind adrenocorticotropic hormone by 5 to 20 min, and in humans, the transfer of cortisol from blood to saliva through diffusion takes place within no more than 2 to 3 min [27]. On the other hand, salivary alpha-amylase is produced by the salivary glands and serum alpha-amylase is mostly produced and released by the pancreas; serum and salivary alpha-amylase are two distinct isoforms [28]. Furthermore, the results from the present study suggest that the effect of pain-related psychological stress on salivary and serum alpha-amylase levels may be different. It has been suggested in several studies that the activity of salivary alpha-amylase is related to psychological triggers [29–31], and one study showed that psychological challenges did not lead to an elevation in blood alpha-amylase levels [32]. For these reasons, the measurement of salivary alpha-amylase levels has certain advantages over the measurement of serum alpha-amylase levels since salivary alpha-amylase levels can effectively reflect the psychological aspect of pain-related stress; additionally, unlike venipuncture, which must be performed for the evaluation of serum alpha-amylase levels, the method used for the measurement of salivary alpha-amylase levels is not invasive.

A consistent decrease in serum cortisol levels after surgical intervention was observed in both groups in the present study, which suggests that serum cortisol levels can be used as indicators of pain-related stress. However, the use of saliva samples for stress evaluation has several advantages over the use of blood samples. Performing venipuncture can induce acute stress and cause an increase in salivary alpha-amylase levels [33]. Saliva sampling can be performed through a non-invasive procedure without causing needle-induced pain or physical strain in patients. Such sampling is easy, repeated collection is possible, and it can be performed by personnel with minimal technical training. More importantly, since the results of the correlation analysis performed in the present study showed that there was a weak positive correlation between salivary alpha-amylase levels and pain scores in dogs who underwent surgery [34], the findings suggest that the level of salivary alpha-amylase can be used as a surrogate indicator or useful biomarker for the assessment of pain-related stress. However, in veterinary practice, there are certain obstacles to the measurement of salivary alpha-amylase levels for the evaluation of stress. Although portable clinical instruments have been used in human clinical practice for the measurement of salivary amylase activity with high accuracy and speed [3, 35], in the field of veterinary science, there is a lack of a high-sensitivity alpha-amylase detection system in dogs; thus the time taken to obtain results is relatively long.

The results of the present study suggest that severe disease-related pain may cause increased psychological stress in patients, which could have caused increased serum and salivary alpha-amylase levels in the high-pain-score group. Furthermore, salivary alpha-amylase levels reflect the activity of the SAM system during pain-induced stress.

Cardiovascular measurements, such as HR and BP measurements, reflect sympathetic activity. However, in the present study, the dogs’ HR and BP did not differ before and after they underwent surgery. These parameters can be affected by many external and internal factors, such as transportation, environmental changes, and emotional factors [36, 37]. Because assessment in the present study was performed in a clinical setting, factors such as exposure to new people, the sight of different animals, and the administration of fluid or drugs could have affected HR and BP. To measure BP, a BP cuff and the oscillometric method were used in this study. Although the oscillometric method is suitable for use in a clinic, it cannot be used to measure average BP. This disadvantage of the oscillometric method may have affected the findings of this study because BP variability is usually quite significant [38].

The present study showed that there was a significant correlation between serum and salivary cortisol levels, and the post-operative decrease in serum cortisol levels was also significant. This result is consistent with the results of previous studies [39–41]. Circadian rhythms associated with salivary and serum cortisol levels in humans have been reported in several studies [42]. However, it remains controversial whether salivary cortisol and serum cortisol levels in dogs show circadian rhythms. It was reported in one study that in dogs, salivary cortisol secretion did not have a circadian rhythm [43]. Another study demonstrated that salivary cortisol follows the circadian rhythm of serum cortisol, and there is a positive correlation between salivary cortisol and serum levels in dogs [40]. In the present study, time was not considered when sampling was performed.

This study has certain limitations. First, to definitively identify the relationship between stress and salivary biomarkers, a study population that is larger than the population included in this study is required. Second, stress-producing factors, such as hospitalization and physical examination, could not be controlled for in this study. To evaluate the use of salivary biomarkers for the assessment of treatment, further studies should be conducted with continuous measurements and considering sample collection times and stress generating factors. To the best of our knowledge, the present study is the first study in which salivary biomarkers and pain parameters in dogs undergoing surgery were evaluated and compared.

## Conclusions

In conclusion, among the dogs that underwent surgery, there was a correlation between salivary alpha-amylase levels and pain scores, and in the high-pain-score group, with a post-operative decrease in pain-related stress, there was a post-operative decrease in salivary alpha-amylase levels. Therefore, salivary alpha-amylase may be a useful biomarker of stress in diseased dogs and a suitable index for the objective assessment of pain intensity.

## Methods

### Animals and study design

All dogs in the study were client-owned, and the owners were informed prior to their inclusion in this study. Dogs were treated at the Veterinary Medical Teaching Hospital College of Chungnam National University and the Time Small Animal Medical Center between August 2018 and July 2019. Cases involving both orthopedic and soft tissue surgery were included. Preoperative sampling was performed on all dogs prior to analgesia and anesthesia. During the operation, dogs were premedicated with intravenous (IV) midazolam 0.2 mg/kg and butorphanol 0.2 mg/kg and anesthesia was induced with propofol 2–6 mg/kg. After intubation, anesthesia was maintained by means of inhalation of isoflurane at 1.5 minimum alveolar concentration (MAC). After the surgery, dogs were given analgesics with constant rate infusion (CRI) of remifentanil hydrochloride 0.1–0.3 mcg/kg/min IV or tramadol-lidocaine-ketamine IV (tramadol 0.1–1.3 mg/kg/hr.; lidocaine 0.6–3.0 mg/kg/hr.; ketamine 0.12–1.2 mg/kg/hr) or bolus of butorphanol 0.1–0.2 mg/kg IV according to pain intensity of dog. CMPS-SF and vital sign checking was used to decided withdrawal of postoperative analgesia. After 7 days of operation, pain and physical examination were re-evaluated and saliva and blood were collected.

### Physical examination and subjective pain assessment

After the initiation of routine physical examination, including the measurement of HR and BP, pain was scored using the CMPS-SF. HR was recorded by stethoscope for 15 s and then multiplying by 4 to determine beats per min. Systolic blood pressure was obtained by the indirect oscillometric method. BP cuffs were sized to 40% of the circumference of the forelimb distal to the elbow of dogs. In the CMPS-SF, with scores ranging from a score of 0 (no pain) to a maximum score of 24, the following five categories associated with behavior were considered: vocalization, attention to wound, response to touch, demeanor, and combined posture/activity. Because orthopedic patients had mobility issues, their mobility was not assessed (section B in the CMPS-SF was not considered). The total score ranges from a score of 0 (which indicates that there is no pain) to a maximum possible score of 20 for orthopedic patients and 24 for other patients. Based on their preoperative CMPS-SF scores, the dogs with > 9 pre-operative CMPS-SF score were included in a high-pain-score group and the dogs with ≤9 pre-operative CMPS_SF score were allocated to low-pain-score group. Physical examinations and the determination of CMPS-SF scores were performed before surgery and 7 days after surgery by the same veterinarian.

### Saliva and blood sampling

The dogs were fasted for more than one hour prior to sampling. After physical examination and determination of CMPS-SF score, dogs were rested for 30 min, and salivary sampling was performed. Saliva specimen was collected using small cotton roll around the mouth for about 3 min. The cotton roll was transferred into 1.5 mL Eppendorf tube and immediately centrifuged at *1.500 g* for 15 min at room temperature. The cotton roll was subsequently removed, and saliva was stored at − 80 °C until analysis. After saliva collection, blood sample was collected by jugular venipuncture into plain tube and the tube was centrifuged at *3.000 g* for 10 min at room temperature. The obtained serum was transferred into Eppendorf tube and stored at − 80 °C until analysis.

### Alpha-amylase and cortisol analysis

Concentrations of AA and cortisol were measured in saliva and serum samples using commercially available enzyme-linked immunosorbent assay kit (alpha-amylase; MyBioSource, USA and cortisol; Salimetrics, USA). The undiluted samples were assayed. The limit of detection for AA analysis was 1.0 ng/mland cortisol analysis was 0.007mcg/dl.

### Statistical analysis

All data were expressed as mean and standard deviation. The Kolmogorov-Smirnov test determined the normal distribution of the data. Comparisons of values before and after surgery were analyzed statistically using the paired t-test. The associations between the CMPS-SF and other parameters (HR, BP, salivary AA, salivary cortisol, serum AA and serum cortisol) were investigated with Pearson’s correlation. Pearson’s correlation was used to analyze the associations between saliva and serum. A *p* value < 0.05 was considered significant. Statistical analyses were performed with GraphPad Prism 9.1 (GraphPad Software, Inc., SanDiego, CA).

## Supplementary Information

**Additional file 1: Supplementary Figure S1**. In both the high-pain-score and low-pain-score groups, there was a correlation between salivary alpha-amylase levels and pain scores. (A, B) Scatter plot between pain score with salivary a-amylase from high pain score group (A) and less pain score group (B). *p* value < 0.05 was considered significant.

**Additional file 2: Supporting Figure S2**. An association between serum cortisol levels and pain scores in the high-pain-score group was observed; however, the correlation was not statistically significant. *p* value < 0.05 was considered significant.

## Data Availability

The datasets used and/or analyzed during the current study available from the corresponding author on reasonable request.

## Abbreviations

AA: alpha-amylase
BP: Blood pressure
CMPS-SF: Short form of the Glasgow composite measure pain scale
HPA: Hypothalamic-pituitary-adrenal
HR: Heart rate
SAM: Sympathetic-adreno-medullary
