# Supplementary figures and images for: Assessment of salivary alpha-amylase and cortisol as a pain related stress biomarker in dogs pre-and post-operation
Source: BMC Vet Res. 2022 Jan 13;18:31. doi: 10.1186/s12917-021-03114-2 (PMC8756664; doi:10.1186/s12917-021-03114-2)

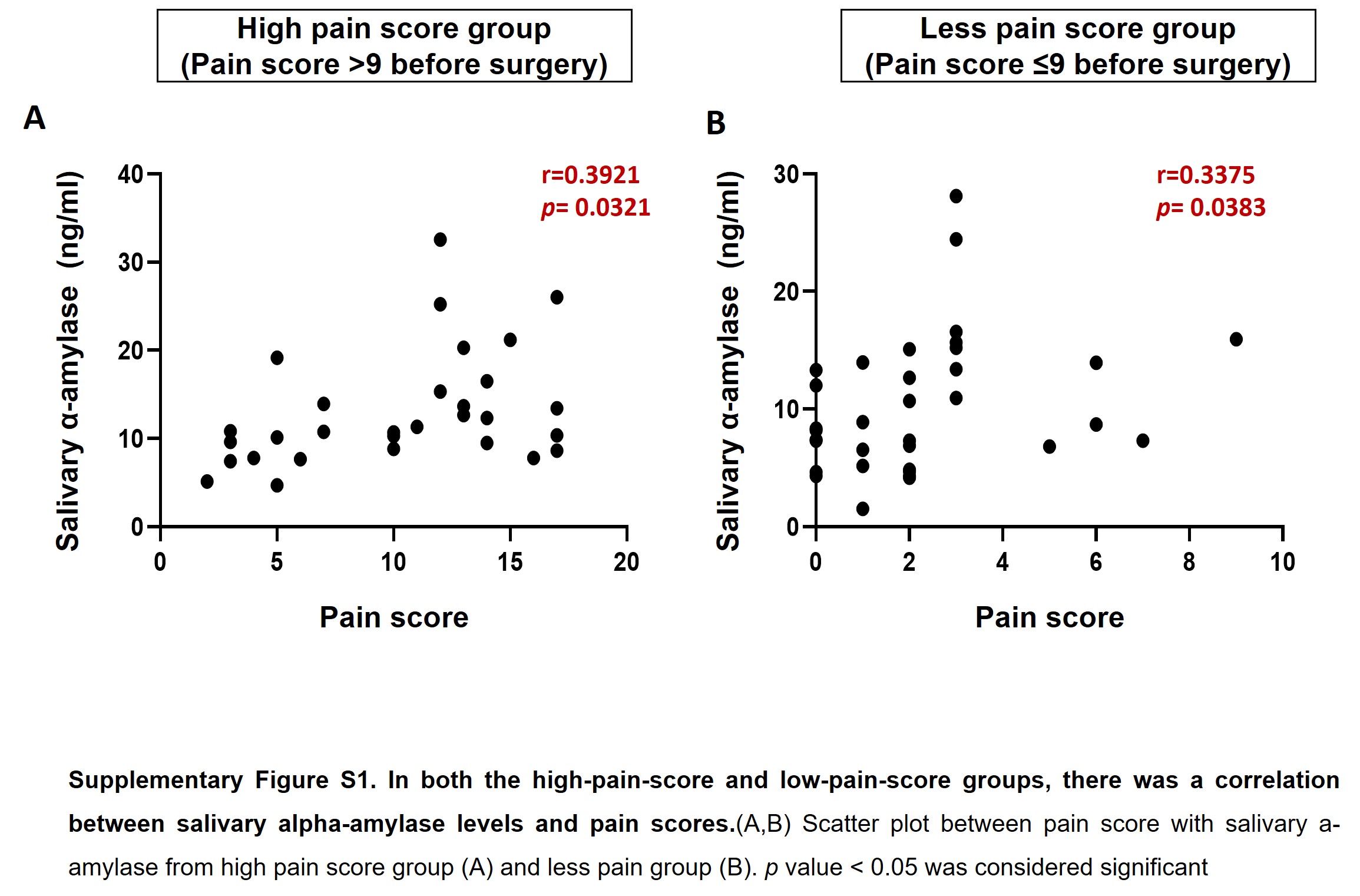

Supplement: Supplementary file 1 — Additional file 1: Supplementary Figure S1. In both the high-pain-score and low-pain-score groups, there was a correlation between salivary alpha-amylase levels and pain scores. (A, B) Scatter plot between pain score with salivary a-amylase from high pain score group (A) and less pain score group (B). p value < 0.05 was considered significant. [file 12917_2021_3114_MOESM1_ESM.jpg]

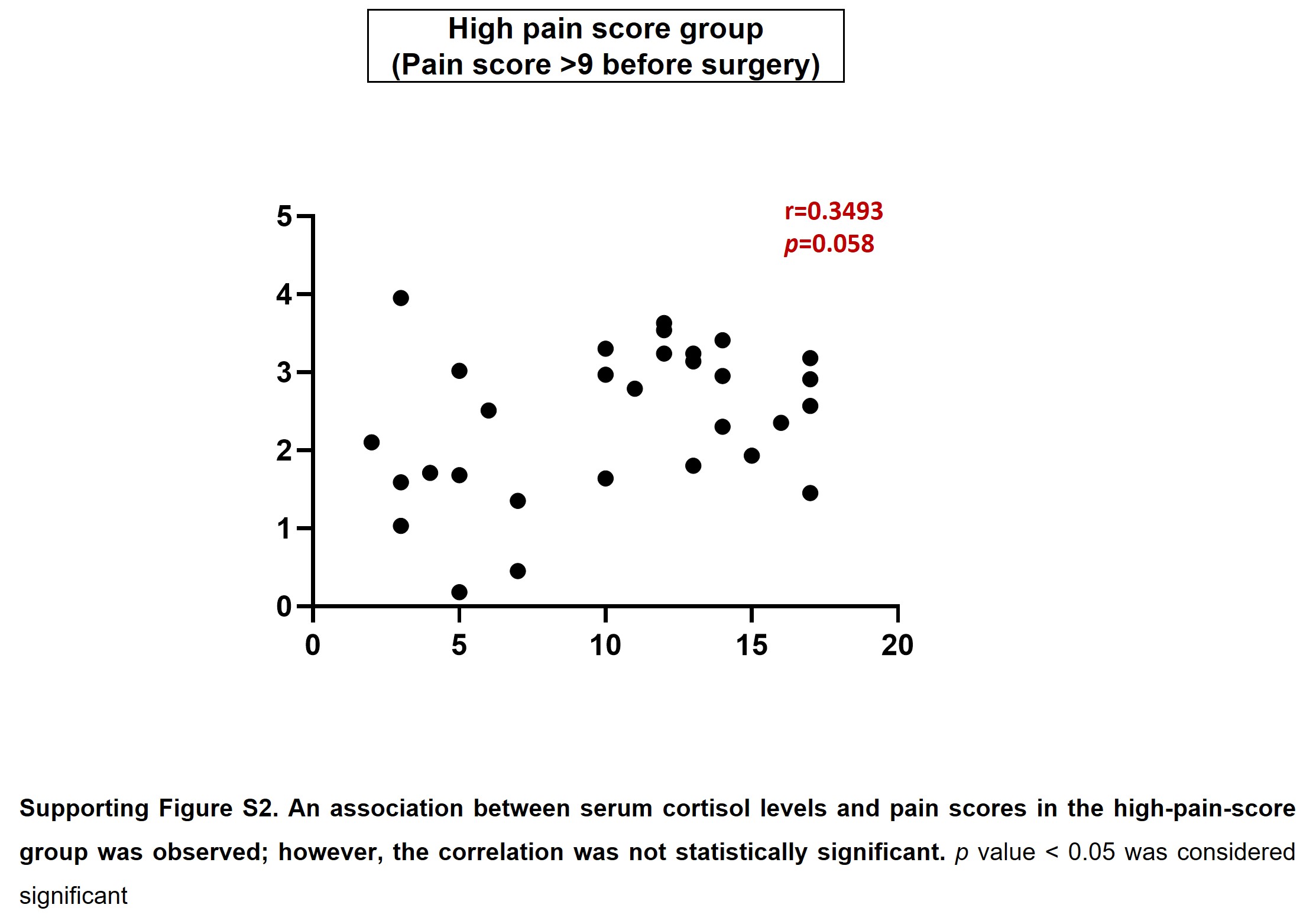

Supplement: Supplementary file 2 — Additional file 2: Supporting Figure S2. An association between serum cortisol levels and pain scores in the high-pain-score group was observed; however, the correlation was not statistically significant. p value < 0.05 was considered significant. [file 12917_2021_3114_MOESM2_ESM.jpg]
